# Bi-allelic mutation of *CTNNB1* causes a severe form of syndromic microphthalmia, persistent foetal vasculature and vitreoretinal dysplasia

**DOI:** 10.1186/s13023-022-02239-3

**Published:** 2022-03-04

**Authors:** Rachel L. Taylor, Carla Sanjuro Soriano, Simon Williams, Denisa Dzulova, Jane Ashworth, Georgina Hall, Theodora Gale, I. Christopher Lloyd, Chris F. Inglehearn, Carmel Toomes, Sofia Douzgou, Graeme C. Black

**Affiliations:** 1 Division of Evolution and Genomic Sciences, School of Biological Sciences, Faculty of Biology Medicine and Health, The University of Manchester, Manchester, UK; 2 Manchester Centre for Genomic Medicine, St Mary’s Hospital, Manchester University NHS Foundation Trust, Manchester Academic Health Sciences Centre (MAHSC), 6Th Floor St Mary’s Hospital, Oxford Road, Manchester, M13 9WL UK; 3 Leeds Institute of Molecular Medicine, St. James’s University Hospital, Leeds, UK; 4 Inserm, Institute for Neurosciences of Montpellier, University of Montpellier, Montpellier, France; 5 Manchester Royal Eye Hospital, Manchester University NHS Foundation Trust, Manchester, UK; 6 Paediatric Ophthalmology, Great Ormond Street Hospital for Children, London, UK; 7 Department of Medical Genetics, Haukeland University Hospital, Bergen, Norway

**Keywords:** *CTNNB1*, Beta-catenin, Recessive, Syndromic, Microphthalmia, Vitreoretinal dysplasia, Developmental delay

## Abstract

**Background:**

Inherited vitreoretinopathies arise as a consequence of congenital retinal vascularisation abnormalities. They represent a phenotypically and genetically heterogeneous group of disorders that can have a major impact on vision. Several genes encoding proteins and effectors of the canonical Wnt/β-catenin pathway have been associated and precise diagnosis, although difficult, is essential for proper clinical management including syndrome specific management where appropriate. This work aimed to investigate the molecular basis of disease in a single proband born to consanguineous parents, who presented with microphthalmia, persistent foetal vasculature, posterior lens vacuoles, vitreoretinal dysplasia, microcephaly, hypotelorism and global developmental delay, and was registered severely visually impaired by 5 months of age.

**Methods:**

Extensive genomic pre-screening, including microarray comparative genomic hybridisation and sequencing of a 114 gene panel associated with cataract and congenital ophthalmic disorders was conducted by an accredited clinical laboratory. Whole exome sequencing (WES) was undertaken on a research basis and in vitro TOPflash transcriptional reporter assay was utilised to assess the impact of the putative causal variant.

**Results:**

In the proband, WES revealed a novel, likely pathogenic homozygous mutation in the cadherin-associated protein beta-1 gene (*CTNNB1),* c.884C>G; p.(Ala295Gly), which encodes a co-effector molecule of the Wnt/β-catenin pathway. The proband’s parents were shown to be heterozygous carriers but ophthalmic examination did not detect any abnormalities. Functional assessment of the missense variant demonstrated significant reduction of β-catenin activity.

**Conclusions:**

This is the first report of a biallelic disease-causing variation in *CTNNB1*. We conclude that this biallelic, transcriptional inactivating mutation of *CTNNB1* causes a severe, syndromic form of microphthalmia, persistent foetal vasculature and vitreoretinal dysplasia that results in serious visual loss in infancy.

**Supplementary Information:**

The online version contains supplementary material available at 10.1186/s13023-022-02239-3.

## Background

Heritable vitreoretinopathies are characterized by abnormal development of the retinal vascular with pathological consequences that have a severe impact on vision, including retinal neovascularization, detachment, exudation, and hemorrhage. Isolated forms can have variable impact on vision, even within families; presentation and phenotypic severity can be gene and mutation dependent. Syndromic forms exist: extra-ocular features may include skeletal, neurological and phycological issues, with differential diagnoses such as Norrie disease, Stickler syndrome, and Wagner syndrome. Whilst clinical phenotyping can help to narrow possible causes, diagnosis is complicated by variable patterns of inheritance, as well as phenotypic and genetic heterogeneity, making identification of the precise cause extremely difficult. The Wnt signalling pathway is crucial for cell migration, survival, differentiation and proliferation. Transcriptional changes orchestrated by Wnt signalling are responsible for organogenesis and angiogenesis, including within the eye and specifically, formation of the complex vascular network that supplies the retina. Defective Wnt signalling, is a predominant underlying cause of familial exudative vitreoretinopathy retinopathy (FEVR) which is characterised by anomalous retinal vascularization. Genes encoding components of the Wnt/β-catenin pathway, NDP (MIM: 300658), FZD4 (MIM: 604579), TSPAN12 (MIM: 61313) and LRP5 (MIM: 603,506), account for a high proportion of diagnoses. The cadherin-associated protein beta-1 (*CTNNB1*) gene (MIM: 116806) which encodes beta-catenin 1 (β-catenin), an integral co-effector of the Wnt/β-catenin pathway and crucial component of the E-cadherens-based adherens junctions, is the most recently associated gene and consequently, its’ phenotypic spectrum an range of variant pathogenicity in relation to vitreoretinal degeneration, is least well understood.

β-catenin, is a highly conserved integral effector molecule of the Wnt/β-catenin pathway. In the absence of Wnt, β-catenin is not ubiquitinated resulting in its cytoplasmic accumulation and translocation in to the nucleus where TCF/LEF transcription factors serve as its main binding partners for the activation of Wnt-responsive genes. β-catenin is also serves a function as a scaffold molecule; immobilised at the adherens junctions by E-cadherin, β-catenin plays an integral role in cadherins-based cell–cell connections through its many interaction partners including α-catenin, via which it may indirectly modulate the actin cytoskeleton. In this way, β-catenin contributes to the polarization of epithelial tissues required for organismal integrity. These roles in cell signalling and adhesion place β-catenin as a key protein in diverse processes including human embryogenesis, adult tissue homeostasis and tumorigenesis [1]. The importance of normal beta-catenin function is reflected in the range and severity of disease phenotypes associated with *CTNNB1* pathogenic variation. Somatic mutations tend to result in gain-of-function, enabling β-catenin to escape degradation, leading to a well-documented role for *CTNNB1* in several different cancers [2]. Heterozygous germline mutations generally result in loss-of-function and result in a spectrum of multi-tissue disease due to *CTNNB1* haploinsufficiency. These include intellectual disability, autism-spectrum disorder, neurodevelopmental disease, neuromuscular disease and craniofacial abnormalities [3–6]. A recent survey of the literature found that approximately 50% of such *CTNNB1* mutations are associated with ophthalmic abnormalities including familial exudative vitreoretinopathy spectrum (FEVR), visual field defects, retinal detachment, strabismus, hyperopia, and lens and vitreous opacities [7–9].

**Fig. 1:**
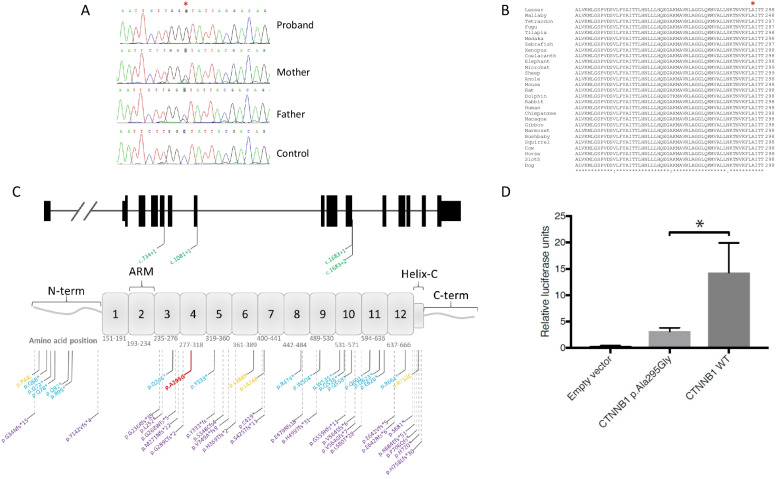
Localisation, conservation and transcriptional activity of *CTNNB1* c.884C>G; p.(Ala295Gly). **A** Sanger sequencing chromatograms confirming the presence and zygosity of the CTNNB1 c.884C>G; p.(Ala295Gly) variant in proband and parental DNA samples, in comparison to a control chromatogram which does not contain the sequence change. **B** *CTNNB1* (NM_001904) gene (top) and β-catenin protein (bottom) schematics annotated with previously reported dominant disease-causing mutations (as reported by HGMDpro; orange text = missense mutations, green text = splice-altering mutations; blue text = nonsense mutations; purple text = small insertion, deletion or insertion/deletion mutations) and the location of the *CTNNB1* c.884C>G; p.(Ala295Gly) homozygous variant identified by this study (indicated by red text). The biallelic missense mutation identified herein resides within the fourth armadillo (ARM) domain of the encoded protein. **C** Multispecies alignment of β-catenin (human amino acids 239–298) showing 100% conservation across 28 species of the alanine residue at position 295, found to be mutated to Glycine in the proband described in this report. Red asterisk (*) above the alignments indicates the position of human Alanine 295 in the alignment. Amino acid residues that are fully conserved are represented by an asterisk underneath the alignments; conservation between residues with strongly similar properties are represented by a colon (:); conservation between residues with weakly similar properties are represented by a period symbol (.). **D** Functional assessment of p.(Ala295Gly) mutant β-catenin on transcription. Bar chart detailing TOPflash luciferase assay results of STF cells transiently co-transfected with wildtype (WT) or mutant (p.(Ala295Gly)) β-catenin or empty vector (pDEST40) and renilla luciferase plasmid. Luciferase activity was measure 48 h post-transfection. Each bar indicates the recorded luciferase activity for each construct following normalisation to renilla activity and relative to the measurement recorded for cells transfected with empty vector. Results are from three independent experiments performed in triplicate. Error bars denote standard error of the mean (SEM); Y-axis represents the fold difference relative to the observed empty vector reading

To-date, 55 different *CTNNB1* variants have been reported as disease-causing, all of which are heterozygous de novo or dominantly inherited mutations (Fig. 1). Herein, we report the first biallelic mutation in *CTNNB1* identified by whole exome sequencing (WES) in a proband with a severe syndromic FEVR-like phenotype of microphthalmia, persistent foetal vasculature, posterior lens vacuoles, and vitreoretinal dysplasia, with extra-ocular features of microcephaly, hypotelorism and global developmental delay. Her parents were apparently unaffected. Assessment of this mutant in vitro demonstrates significantly reduced transcriptional activation, providing an insight in to the potential effect of the variant on normal β-catenin function.

## Results

### Case report

The proband, a female, was the first-born child to first cousin parents originating from Bangladesh. There was no known family history of medical problems. The proband was delivered at 42 weeks by caesarean section due to non-progressing labour, weighing 3.7 kg. Her parents noticed poor visual behaviour shortly thereafter. Examination by a paediatric ophthalmologist aged 4 months diagnosed roving eye movements. She demonstrated an ability to fix and follow with both eyes open but not when tested uniocularly. She had a large angle esotropia of sensory origin, bilateral severe microphthalmia. She underwent examination under anaesthetic and horizontal corneal diameters were 7.0 mm right and 7.5 mm left (mean diameter for normal eyes at 1 day- 6 months = 11.43 mm ± 0.59 mm sd) and axial length 10.6 mm left (mean length for normal eyes at 3–6 months = 19.76 mm ± 0.62 mm sd). Measurement of axial length was not possible in the right eye. She had bilateral persistent foetal vasculature (PFV) with persistent hyaloid arteries, and posterior lens vacuoles on the left. Optic discs were full and dysplastic optic discs and retinae were flat with a ‘glazed’ appearance. Electrodiagnostic testing showed flash response to visual evoked potentials (VEP) and a generalised abnormal electroretinogram (ERG) (i.e.- namely reduction of amplitude of oscillatory potentials, a and b wave of flash ERG, scotopic and photopic b waves). She was registered severely visually impaired at 5 months of age. Aged 7 months, she underwent left lensectomy and an anterior segment revision four weeks later. At this time, ultrasound of the right eye detected right tractional retinal detachment, dense cataract, and no visual potential. By the age of 4 years, her right eye had become phthisical with band keratopathy. At her most recent ophthalmic assessment aged 6 years, her left visual acuity measured 1.6 Crowded Kay’s, and no light perception in the right. Fundus examination of the proband’s mother (under dilation) and father (not dilated) did not detect any abnormalities.

Evaluation at 6 months of age by a clinical geneticist found she was growing normally in terms of height and weight, but head circumference was on the lower normal range. Developmentally, she did not roll on either side by 5 months; she sat on her own at 9 months and pulled up to stand at 10 months. By 11 months, her height was between the 2nd and 9th centiles, weight on the 25th centile, and OFC < 0.4th centile by at least one standard deviation. There was no eruption of dentition by 11 months of age and she was noted to have distinct facial features compared to her parents, including significant hypotelorism. Magnetic Resonance Imaging (MRI) of the brain at 2.5 years of age was normal. At 3 years of age, height and weight were tracking the 50th centile, while OFC remained below normal range. She was diagnosed with global developmental delay by 5 years of age. At this stage, her gross motor skills were age appropriate, however her fine motor skills and speech were delayed. Her hearing was normal.

### Genomic findings

Cataract Panel NGS and array comparative genomic hybridisation (aCGH) testing did not detect any genomic variants, chromosomal gains or losses that might be accountable for the proband’s presenting phenotype. WES data was analysed for variants in known FEVR genes, including the possibility of complex/digenic inheritance as has been previously reported in this phenotype [10–13]. No putative causal variants were identified. Given the consanguineous relationship status of the parents, the proband’s WES data was enriched for rare (MAF ≥ 0.001) homozygous, protein altering variants that had not previously been detected in an in-house dataset of 586 exomes. This analysis identified four candidate variants (see Additional file 1: Data 1), of which the *CTNNB1* c.884C>G; p.(Ala295Gly) homozygous missense variant was considered the most likely cause of disease. Relevant ACMG evidence categories were PM2, PP1, PP2, PP3, PP4, leading to classification of the identified variant as ‘Likely pathogenic’. Heterozygous germline variants in *CTNNB1* have previously been reported as a cause of FEVR, developmental delay and intellectual disability. This variant has not previously been reported in any control cohorts (GnomAD, ExAC, EVS or dbSNP), nor has it previously been reported as disease-causing. The change affects a highly conserved nucleotide in exon 6 of the canonical transcript (NM_001904) that converts a highly conserved Alanine residue to Glycine at positon 295 of the encoded protein, within the fourth Armadillo (ARM) domain (Fig. 1). In silico tools predict this change to be highly damaging to protein function (SIFT: Deleterious (score: 0.01); Polyphen-2: Possibly damaging (score: 0.68) (Supplementary Data 1).

### In vitro assessment of variant impact on protein function

Previous work investigating the effect of *CTNNB1* variants on protein function in vivo and in vitro have demonstrated reduced *Drosophila* armadillo (a β-catenin homolog) signalling and mutation-dependent contrasting effects on β-catenin transcriptional activity when compared to wild-type, respectively [9, 14]. We assessed the impact of the *CTNNB1* c.884C > G; p.(Ala295Gly) variant on β-catenin on transcriptional activity using a TOPflash reporter assay system, as a means of determining the potential mode of pathogenicity. The results showed that the *CTNNB1* c.884C>G; p.(Ala295Gly) variant of β-catenin leads to significantly reduced levels of TOPflash activity compared to wild-type, almost to null allele levels (Fig. 1).

## Discussion

This study describes a novel biallelic mutation in *CTNNB1* and the severe, syndromic ophthalmic phenotype arising from it. The proband experienced complex ophthalmic problems from infancy, including marked microphthalmia, PFV, cataract as well as vitreoretinal dysplasia, that required surgical intervention throughout the early years of her life, culminating in total retinal detachment in her right eye and her registration as severely visually impaired before 7 months of age. She also presented with significant microcephaly, hypotelorism and developmental delay. Following extensive genetic pre-screening, the proband was found to harbour a *CTNNB1* c.884C>G; p.(Ala295Gly) homozygous variant by WES. In vitro characterisation revealed this variant results in very low β-catenin transcriptional activity levels suggesting it is acting as a hypomorphic mutation, resulting in a severe phenotypic presentation in the proband, whilst the heterozygous carrier parents remain apparently unaffected. To the best of our knowledge, this is the first report of disease caused by a recessive *CTNNB1* mutation.

*CTNNB1* encodes the final effector of the Wnt/β-catenin pathway, β-catenin, and is the most recently associated FEVR gene [9, 15]. As with other Canonical/Wnt pathway disease-associated genes, *CTNNB1* mutations result in a range of extra-ocular phenotypes including intellectual disability, developmental and neurological delay. FEVR is clinically and genetically heterogeneous. The condition also demonstrates extensive variability in phenotypic severity, even within families [16], ranging from asymptomatic far peripheral retinal avascularity to total retinal detachment with consequential complete blindness. There is increasing evidence that mutation type, affected gene, expressivity and mutation zygosity all contribute to disease presentation [17]. Mutations in genes involved at every stage of the Canonical/Wnt signalling pathway, have been reported to cause FEVR, including Norrin (*NDP*; a Wnt ligand), Frizzled-4 (*FZD4*; a Wnt receptor), Lipoprotein receptor 5 (*LRP5*, a Wnt receptor), Kinesin family member 11 (KIF11, microtubule motor), and Tetraspanin 12 (*TSPAN12*; Wnt signalling mediator). Mutations in Zinc finger protein 408 (ZNF408)—the function of which is currently unclear- are also known to cause FEVR.

Previous reports of *CTNNB1*-associated ophthalmic abnormalities range from lens and vitreous opacities, strabismus, myopia and hyperopia [4, 18], to retinal detachment [8], and FEVR [9, 15], with or without the aforementioned extra-ocular manifestations. The proband described by this study presented with a phenotype that falls at the severe syndromic end of this disease spectrum, including microcephaly and neurodevelopmental delay, likely due to the demonstrated deleterious effect of the *CTNNB1* c.884C>G; p.(Ala295Gly) homozygous variant. Of the 55 previously reported disease-causing *CTNNB1* variants, the majority (92.7%, n = 51) are predicted to result in premature termination or disruption of the encoded protein. Only 7.3% (n = 4) of reported pathogenic variants are missense mutations. Each of the four missense variants have been reported in association with a different phenotypic presentation, including bone dysplasia and adrenocortical adenoma [19], complex developmental phenotypes involving delayed motor and neurological development with craniofacial dysmorphism [4, 20], and isolated FEVR [9]. Two of these mutations have been assessed functionally via in vitro luciferase assay and shown to result in increased β-catenin activity [9, 19]. In contrast, in vitro characterisation of the *CTNNB1* c.884C>G; p.(Ala295Gly) mutation identified by our analysis using the same or similar luciferase chemistry, detected significantly reduced β-catenin activity indicating it is likely a hypomorph. It is difficult to speculate on the possible mechanisms responsible for these contradictory findings, however similar contrasting outcomes have been detected by TOPflash assays measuring the effects of *NDP* variants [21, 22]. These assays are unlikely to precisely replicate the effect in humans since the molecular mechanisms controlling β-catenin expression are very complex, but do represent an effective means of identifying variants with an impact on protein function. Further work to decipher the different effects of *CTNNB1* missense variants is an important area of future research, especially in the development of treatments. Reduction of β-catenin transcription has been shown to result in defective angiogenesis and maintenance of blood–brain and blood-retina barriers in mouse knock-out models [23, 24], whilst overexpression of Norrin/β-catenin signalling causes disrupted embryonic angiogenesis [25]. In mice, conditional knock-out of β-catenin results in a phenotype that includes microphthalmia, cataract, and eye spacing abnormalities, similar to the patient described here [26]. A small number of disease-causing *CTNNB1* missense variants have been reported to date: evaluation of potential genotype–phenotype correlations remains an important area for further research.

A number of FEVR-associated genes are well documented to cause both autosomal dominant and recessive disease (i.e. *FZD4*, *LRP5*, and *TSPAN12*). *CTNNB1* has previously been reported as a cause of dominant disease. Our research now provides evidence that *CTNNB1* may also cause recessive disease with severe phenotypic severity. Interestingly, research has shown that on rare occasions, some phenotypic variability within FEVR families can be attributed to zygosity for the same mutation, i.e.—family members with two mutant alleles may be more severely affected than those carrying one copy of the same mutant allele [25]. The sensitivity of FEVR to gene dosage is not very well understood due to the scarcity of reported recessive cases and difficulties in diagnosing, often visually asymptomatic, mild cases. However, it is tempting to speculate, given the broad phenotypic spectrum of documented dominant FEVR cases, at least some severe cases may be due multiple variants, either in the same or different FEVR genes. This represents an important area for future research, including a possible role for genetic modifiers, that may be common in the general population, in moderating the severity of FEVR presentations. The parents of the proband described in this study were confirmed to be heterozygous carriers of the *CTNNB1* c.884C>G; p.(Ala295Gly) variant. They were found to be visually asymptomatic and fundus examination was unremarkable although wide-field fluorescein angiography to rule out far peripheral avascularity would be required before the status of the carrier parents can be conclusively confirmed.

## Conclusion

We provide the first report of biallelic mutation in *CTNNB1* in a proband with a severe syndromic FEVR phenotype. The identified *CTNNB1* c.884C>G; p.(Ala295Gly) homozygous variant has not previously been reported as disease-causing in heterozygous state, nor has it been detected in control cohorts. Our analysis has shown that this mutation produces a gene product with severely reduced levels of functional activity. Our work demonstrates that future discovery of further hypomorphic alleles and timely diagnosis of associated complex or ambiguous syndromic ophthalmic phenotypes, requires astute selection of genetic test by the referring clinician following clinical phenotyping. As further genes and variants underlying FEVR phenotypes are identified, a better understanding of the disease spectrum will be gained, facilitating diagnostic testing and therapeutic development.

## Methods

### Ethics and patient recruitment

The study participant was recruited at Manchester Centre for Genomic Medicine as part of an ongoing study of inherited retinal degeneration in families without a molecular diagnosis following NGS screening for a panel of known genes (The UK Inherited Retinal Disease Consortium). Ethics committee approval was obtained for all aspects of this study (11/NW/0421 and 15/YH/0365) and the protocol observed the tenets of the Declaration of Helsinki. Written informed consent was obtained from the parents on behalf on the proband due to her young age, as an essential pre-requisite for study inclusion.

### Clinical assessment

The participant underwent full ophthalmic assessment by a paediatric ophthalmologist, including visual acuity and dilated fundus examination whilst under anaesthetic. Mother of the proband also underwent fundus examination under dilation. Electroretinography (ERG) was performed in the proband to standards specified by the International Society for Clinical Electrophysiology of Vision (ISCEV). Family history, developmental and dysmorphology assessments were conducted by a clinical geneticist.

### Genomic analyses

#### Array comparative genomic hybridisation (aCGH)

aCGH was performed at Manchester Centre for Genomic Medicine using the CytoSure™ Constitutional v3 8 × 60 k microarray (Oxford Gene Technologies, Oxford, UK), according to manufacturers’ instructions.

#### Cataract NGS panel analysis

DNA from the proband also underwent Cataract Panel NGS for114 genes associated with paediatric cataract and associated lenticular and ophthalmic developmental abnormalities using a custom-designed SureSelectXT target enrichment (Agilent, Santa Clara, Ca, USA) and HiSeq 2500 (Illumina, Inc. San Diego, Ca, USA) sequencing, as previously described (27).

#### Whole exome sequencing

DNA libraries for WES were prepared using 200 ng of high quality DNA. Exons and 50 bp of flanking intronic sequence were captured using the SureSelect Human All Exon V6 Enrichment kit (Agilent) for HiSeq 2500 (Illumina) sequencing. Sequencing reads were aligned using bwa (v0.7.12), and processed for duplicates, in/del realignment using a combination of Samtools, Picard and the Genome Analysis Toolkit (GATK), according to ACGM best practice guidelines. Variant calling was conducted using GATK (version 3.3-0) and annotated using ANNOVAR, based on Ensembl v75 genes and transcripts.

#### Variant interpretation

Variant filtering was performed using MAF in publicly available and in-house datasets, predicted protein impact and familial segregation. Surviving variants were prioritized based on relevance to disease presentation and following extensive appraisal of the scientific literature. Variant interpretation was conducted in broad concordance with the 2015 American College of Medical Genetics and Genomics Best Practice guidelines. In silico modelling predictions from tools such as SIFT, PolyPhen2 and CADD were utilised to support interpretation of variant impact.

### Transcriptional reporter assay

Transcriptional activity of the identified variant was assessed using the TOPflash β-catenin Transcriptional Reporter Assay (Life Technologies, Carlsbad, Ca, USA). Untagged expression constructs for wildtype (WT) or mutant (A295G) in pDEST40 (Life Technologies) were created using the QuikChange II XL site-directed mutagenesis kit (Agilent). The assays were performed in HEK293 cells stably transfected with the TOPflash firefly luciferase construct (also known as STF cells, a kind gift of Jeremy Nathans). Experiments were performed in triplicate in a 24 well plate, and repeated over three independent experiments. 90,000 cells /well were transfected with 399 ng of construct DNA plus 1 ng of *Renilla* luciferase control plasmid (pRL-TK) (Promega, Madison, Wi, USA), using 1.5µL of FuGENE 6 transfection reagent (Promega). Luciferase activity was measured after 48 h using the Dual-Luciferase Reported Assay System (Promega) on the Mithras LB 940 plate reader (Berthold Technologies, Harpenden, UK). The data was analysed by one-way ANOVA and Dunnett’s test using GraphPad PRISM 7.0 software.

## Supplementary Information

**Additional file 1: Data 1.** Candidate variants arising from whole exome sequencing analysis. Table details the variants resulting following filtering steps and details of how each variant was interpreted according to guidelines published by the American College of Medical Genetics and Genomics and the Association for Molecular Pathology (Richards et al., 2015). Chr: chromosome; Hom: homozygous; HGVS: Human Genome Variation Society; EGF-like: epidermal growth factor-like; AF: Allele frequency; MIM: Mendelian inheritance in Man I.D. number. * Evidence and Classifications calculated according criteria from Richards et al., 2015 

## Data Availability

The datasets generated and/or analysed during the current study are not publicly available due to patient privacy but are available from the corresponding author on reasonable request.

## Abbreviations

*CTNNB1*: Cadherin-associated protein beta-1
ERG: Electroretinogram
FEVR: Familial exudative vitreoretinopathy
FZD4: Frizzled-4
HEK293: Human embryonic kidney 293 cell line
ISCEV: International Society for Clinical Electrophysiology of Vision
LRP5: Lipoprotein receptor 5
NDP: Norrin
OFC: Occipital frontal circumference
TSPAN12: Tetraspanin 12
VEP: Visual evoked potentials
WES: Whole exome sequencing
WT: Wildtype
ZNF408: Zinc finger protein 408
