# Supplementary material for: Bi-allelic mutation of CTNNB1 causes a severe form of syndromic microphthalmia, persistent foetal vasculature and vitreoretinal dysplasia
Source: Orphanet J Rare Dis. 2022 Mar 4;17:110. doi: 10.1186/s13023-022-02239-3 (PMC8896279; doi:10.1186/s13023-022-02239-3)
Supplement: Supplementary file 1 — Additional file 1: Data 1. Candidate variants arising from whole exome sequencing analysis. Table details the variants resulting following filtering steps and details of how each variant was interpreted according to guidelines published by the American College of Medical Genetics and Genomics and the Association for Molecular Pathology (Richards et al., 2015). Chr: chromosome; Hom: homozygous; HGVS: Human Genome Variation Society; EGF-like: epidermal growth factor-like; AF: Allele frequency; MIM: Mendelian inheritance in Man I.D. number. * Evidence and Classifications calculated according criteria from Richards et al., 2015 [file 13023_2022_2239_MOESM1_ESM.pptx]

## Slide 1
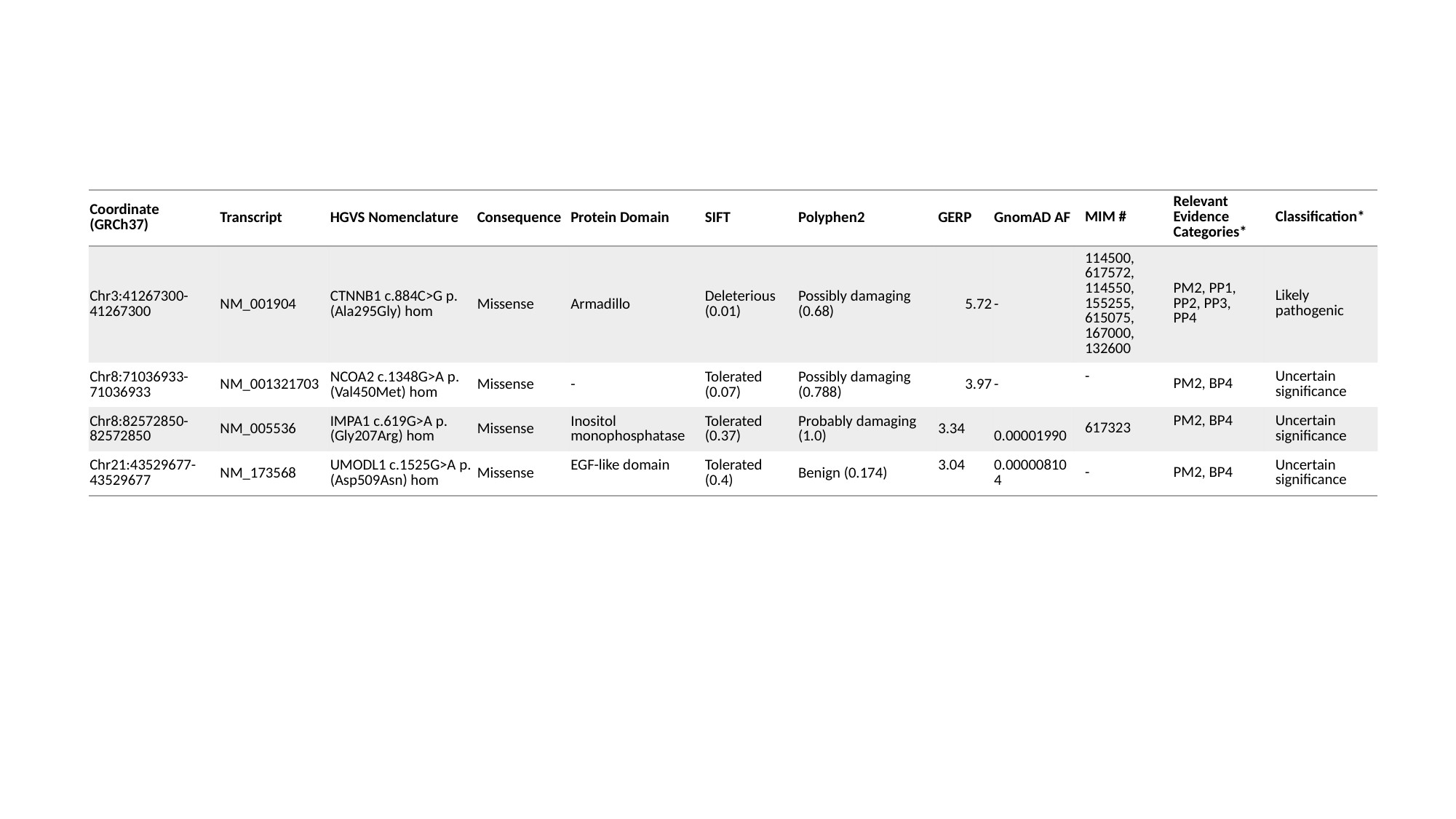

| Coordinate (GRCh37) | Transcript | HGVS Nomenclature | Consequence | Protein Domain | SIFT | Polyphen2 | GERP | GnomAD AF | MIM # | Relevant Evidence Categories\* | Classification\* |
| --- | --- | --- | --- | --- | --- | --- | --- | --- | --- | --- | --- |
| Chr3:41267300-41267300 | NM\_001904 | CTNNB1 c.884C>G p.(Ala295Gly) hom | Missense | Armadillo | Deleterious (0.01) | Possibly damaging (0.68) | 5.72 | - | 114500, 617572, 114550, 155255, 615075, 167000, 132600 | PM2, PP1, PP2, PP3, PP4 | Likely pathogenic |
| Chr8:71036933-71036933 | NM\_001321703 | NCOA2 c.1348G>A p.(Val450Met) hom | Missense | - | Tolerated (0.07) | Possibly damaging (0.788) | 3.97 | - | - | PM2, BP4 | Uncertain significance |
| Chr8:82572850-82572850 | NM\_005536 | IMPA1 c.619G>A p.(Gly207Arg) hom | Missense | Inositol monophosphatase | Tolerated (0.37) | Probably damaging (1.0) | 3.34 | 0.00001990 | 617323 | PM2, BP4 | Uncertain significance |
| Chr21:43529677-43529677 | NM\_173568 | UMODL1 c.1525G>A p.(Asp509Asn) hom | Missense | EGF-like domain | Tolerated (0.4) | Benign (0.174) | 3.04 | 0.000008104 | - | PM2, BP4 | Uncertain significance |
